# Using machine learning for predicting cancer-specific mortality in bladder cancer patients undergoing radical cystectomy: a SEER-based study

**DOI:** 10.1186/s12885-025-13942-2

**Published:** 2025-03-21

**Authors:** Lei Dai, Kun Ye, Gaosheng Yao, Juan Lin, Zhiping Tan, Jinhuan Wei, Yanchang Hu, Junhang Luo, Yong Fang, Wei Chen

**Affiliations:** 1 Department of Urology, The First Affiliated Hospital, Sun Yat-sen University, Guangzhou, 510080 China; 2 Department of Pediatrics, The Third Affiliated Hospital, Sun Yat-Sen University, Guangzhou, 510630 China; 3 Sun Yat-sen University School of Medicine, Guangzhou, 510080 China; 4 Department of Urology, The First Affiliated Hospital of Sun Yat-sen University, 58 Zhongshan Second Road, Guangzhou, Guangdong 510080 China

**Keywords:** Bladder cancer, Radical cystectomy, Machine learning, Prognosis model, SEER

## Abstract

**Background:**

Accurately assessing the prognosis of bladder cancer patients after radical cystectomy has important clinical and research implications. Current models, based on traditional statistical approaches and complex variables, have limited performance. We aimed to develop a machine learning (ML)-based prognostic model to predict 5-year cancer-specific mortality (CSM) in bladder cancer patients undergoing radical cystectomy, and compare its performance with current validated models.

**Methods:**

Patients were selected from the Surveillance, Epidemiology, and End Results database and the First Affiliated Hospital of Sun Yat-sen University for model construction and validation. We used univariate and multivariate Cox regression to select variables with independent prognostic significance for inclusion in the model’s construction. Six ML algorithms and Cox proportional hazards regression were used to construct prediction models. Concordance index (C-index) and Brier scores were used to compare the discrimination and calibration of these models. The Shapley additive explanation method was used to explain the best-performing model. Finally, we compared this model with three existing prognostic models in urothelial carcinoma patients using C-index, area under the receiver operating characteristic curve (AUC), Brier scores, calibration curves, and decision curve analysis (DCA).

**Results:**

This study included 8,380 patients, with 6,656 in the training set, 1,664 in the internal validation set, and 60 in the external validation set. Eight features were ultimately identified to build models. The Light Gradient Boosting Machine (LightGBM) model showed the best performance in predicting 5-year CSM in bladder cancer patients undergoing radical cystectomy (internal validation: C-index = 0.723, Brier score = 0.191; external validation: C-index = 0.791, Brier score = 0.134). The lymph node density and tumor stage have the most significant impact on the prediction. In comparison with current validated models, our model also demonstrated the best discrimination and calibration (internal validation: C-index = 0.718, AUC = 0.779, Brier score = 0.191; external validation: C-index = 0.789, AUC = 0.884, Brier score = 0.137). Finally, calibration curves and DCA exhibited better predictive performance as well.

**Conclusions:**

We successfully developed an explainable ML model for predicting 5-year CSM after radical cystectomy in bladder cancer patients, and it demonstrated better performance compared to existing models.

**Supplementary Information:**

The online version contains supplementary material available at 10.1186/s12885-025-13942-2.

## Introduction

Globally, bladder cancer ranks as the tenth most common cancer, with a higher incidence in males. It is the sixth most prevalent cancer and the ninth leading cause of cancer-related deaths. According to statistics, in 2020, there were approximately 573,000 new cases and 213,000 deaths due to bladder cancer [1]. The most common type of bladder cancer is urothelial carcinoma, which accounts for over 90% of bladder cancer cases. The remaining types include squamous cell carcinoma, adenocarcinoma, and others [2]. Radical cystectomy combined with pelvic lymph node (LN) dissection is the standard treatment for muscle-invasive or high-risk non-muscle invasive urothelial carcinoma of the bladder. In addition, radical cystectomy and LN dissection should also be considered for non-urothelial bladder cancers, such as squamous cell carcinoma and adenocarcinoma of the bladder [3, 4]. The choice between an orthotopic neobladder and a urinary diversion after radical cystectomy depends on factors such as the patient’s age, overall health, and other medical conditions. Older patients generally tend to opt for urinary diversion due to its lower surgical risks, shorter recovery period, and simpler postoperative management [5]. The 5-year cancer-specific mortality (CSM) rate for patients after radical cystectomy is approximately 46% [6]. Predicting the prognosis of patients after surgery is clinically important because those with a poorer estimated survival may be candidates for adjuvant therapy or potential clinical trials and benefit from them.

Many prognostic models for assessing the prognosis of patients after radical cystectomy have been published [7–12], and some have been well validated. However, the use of these models is limited due to the large number of variables involved, making it difficult to obtain the necessary information. Additionally, these models are mostly constructed using traditional methods, which may not fully leverage the advantages of modern technology and data mining techniques [7, 8].

Compared to traditional methods, machine learning (ML) offers a new analytical approach that can automatically learn and improve without explicit programming. By analyzing large amounts of clinical data, ML can assist doctors in predicting disease risk and progression [13]. It has been widely applied in the field of medicine, such as in the diagnosis of occlusive myocardial infarction [14], prediction of acute kidney injury in children [15], and prognosis of non-metastatic prostate cancer [16].

Our objective was to use ML methods, based on the Surveillance, Epidemiology, and End Results (SEER) database, to construct an explainable prognostic model for predicting the 5-year CSM of bladder cancer patients after radical cystectomy, and compared its performance with existing models.

## Methods

### Data source and study population

We used data from the SEER Program to develop our model, which includes approximately 30% of cancer diagnosis and survival data for the American population. Patients diagnosed with histologically confirmed bladder cancer from 2000 to 2020, who were at least 18 years old and underwent radical cystectomy, were included. Patients with extranodal metastases at diagnosis and those lacking accurate and complete information on race, marital status, tumor (T) stage, total lymph node (LN) counts, positive LN counts, metastasis (M) stage, and chemotherapy status were excluded. Complete survival data for patients were required, and the follow-up time for surviving patients had to be at least five years. Access to the SEER database does not necessitate further ethical approval or informed consent beyond signing the SEER Research Data Agreement, as all records within the database are publicly available and de-identified.

The external validation set comprised 60 bladder cancer patients who underwent radical cystectomy at the First Affiliated Hospital of Sun Yat-sen University between 2016 and 2019. Inclusion and exclusion criteria were aligned with those of the SEER database. The final follow-up was conducted in April 2024. Our retrospective cohort study, approved by the Ethics Committee of the First Affiliated Hospital of Sun Yat-sen University, received a waiver for informed consent due to the absence of personally identifiable patient information in the data used for this study.

### Variable processing and selection

This study collected variables including age at diagnosis, sex, race, marital status, pathology, tumor size, grade, T stage, LN density, and chemotherapy. LN density is defined as the ratio of positive LN counts to total LN counts. The missing data in grade (6.5%) and tumor size (23%) were imputed using the multiple imputation method. These variables were subjected to both univariate and multivariate Cox regression analyses to assess patients’ risk of mortality and to identify independent prognostic markers.

### Model development and performance comparison

The data obtained from the SEER was split, allocating 80% for training and 20% for internal validation, ensuring mitigation of potential overfitting issues. Furthermore, the dataset from the First Affiliated Hospital of Sun Yat-sen University was employed for external validation.

The selected variables were used for constructing the prognostic model. Six machine learning models, namely Light Gradient Boosting Machine (LightGBM), Gradient Boosting Decision Tree (GBDT), Extreme Gradient Boosting (XGBoost), Decision Tree (DT), Adaptive Boosting (AdaBoost), K-Nearest Neighbor (KNN), and a traditional model Cox Proportional Hazards regression model (CPH), were used to predict CSM in bladder cancer patients undergoing radical cystectomy. To enhance the prediction model’s performance, a combination of grid search, and ten-fold cross-validation was utilized to determine the final set of hyperparameters. This approach allowed for a comprehensive exploration of the hyperparameter space, ensuring optimal parameter selection while mitigating the risk of overfitting through rigorous model evaluation across multiple folds of the dataset.

Concordance indices (C-indices) were computed to assess model discrimination, while Brier scores were used for calibration evaluation. The c-index indicates the effectiveness of models in discriminating patients’ CSM risk, and calibration evaluates the alignment between predicted and observed outcomes. Additionally, the confusion matrix, a crucial tool for assessing the performance of classification models, was utilized to measure accuracy and precision. Finally, a Kaplan-Meier (K-M) curve was plotted to evaluate the model’s ability to distinguish between surviving and deceased patients.

### Model explanation

The Shapley additive explanations (SHAP) method is a powerful and widely used technique in the field of machine learning and predictive modeling. It is designed to provide a comprehensive and detailed understanding of how each variable within a model contributes to the final prediction. SHAP values are based on the Shapley value concept from cooperative game theory. In the context of our model, it assigns a specific value to each variable, which represents the marginal contribution of that variable to the predicted outcome. One of the key advantages of using SHAP is its ability to handle complex models, such as those incorporating multiple variables and nonlinear relationships. It can break down the black-box nature of advanced machine learning algorithms and present the results in an intuitive and interpretable manner. This is crucial in the medical field as clinicians need to have a clear understanding of why a model makes a certain prediction.

### Head-to-head model comparison

In both the internal and external validation sets, the best-performing model above was compared with three existing prognostic models. The attributes of each model are shown in Supplementary Table S1. These three models consist of the American Joint Committee on Cancer (AJCC) 8th edition stage [17], the Cancer of the Bladder Risk Assessment (COBRA) score [9], and a multivariable model (MTNSC) [11]. Since the COBRA score and the MTNSC model are only applicable to patients with urothelial carcinoma, non-urothelial carcinoma patients were excluded during the comparison process.

The model’s discrimination ability was assessed using the C-index and the receiver operating characteristic (ROC) curve, including the calculation of the area under the curve (AUC). The alignment between predicted and observed outcomes was evaluated through the Brier score and calibration curve. Moreover, decision curve analysis (DCA) was applied to compare the threshold probability ranges of each model, assessing their clinical utility and benefits.

### Statistical analysis

We adopted the multiple imputation method to handle missing data and utilized six machine learning algorithms, including LightGBM, GBDT, XGBoost, DT, AdaBoost, and KNN, along with the CPH model to construct the prediction models. The hyperparameters of the models were optimized through grid search and ten-fold cross-validation. To interpret the final model, we employed the SHAP method to calculate the contribution of each variable to the prediction result. The positive and negative SHAP values indicate the direction of the feature’s impact on the prediction outcome.

The discriminatory ability of the models was evaluated by the C-index and the ROC curve (with the AUC calculated). Calibration ability was assessed using the Brier score and the calibration curve, where a calibration curve closer to the diagonal indicates better calibration. To compare the clinical utility of the models, DCA was adopted to assess the clinical value by calculating the net benefit at different thresholds. Additionally, we evaluated the accuracy and precision of the classification models using the confusion matrix and plotted the Kaplan-Meier (K-M) curve to assess the model’s ability to distinguish between surviving and deceased patients.

All statistical analyses were conducted using R version 4.3.1 (https://www.r-project.org) and Python version 3.9.18 (https://www.python.org), with the relevant packages listed in Supplementary Table S2. Statistical significance was set at a two-tailed P-value of less than 0.05.

## Results

### Patient characteristics

**Fig. 1:**
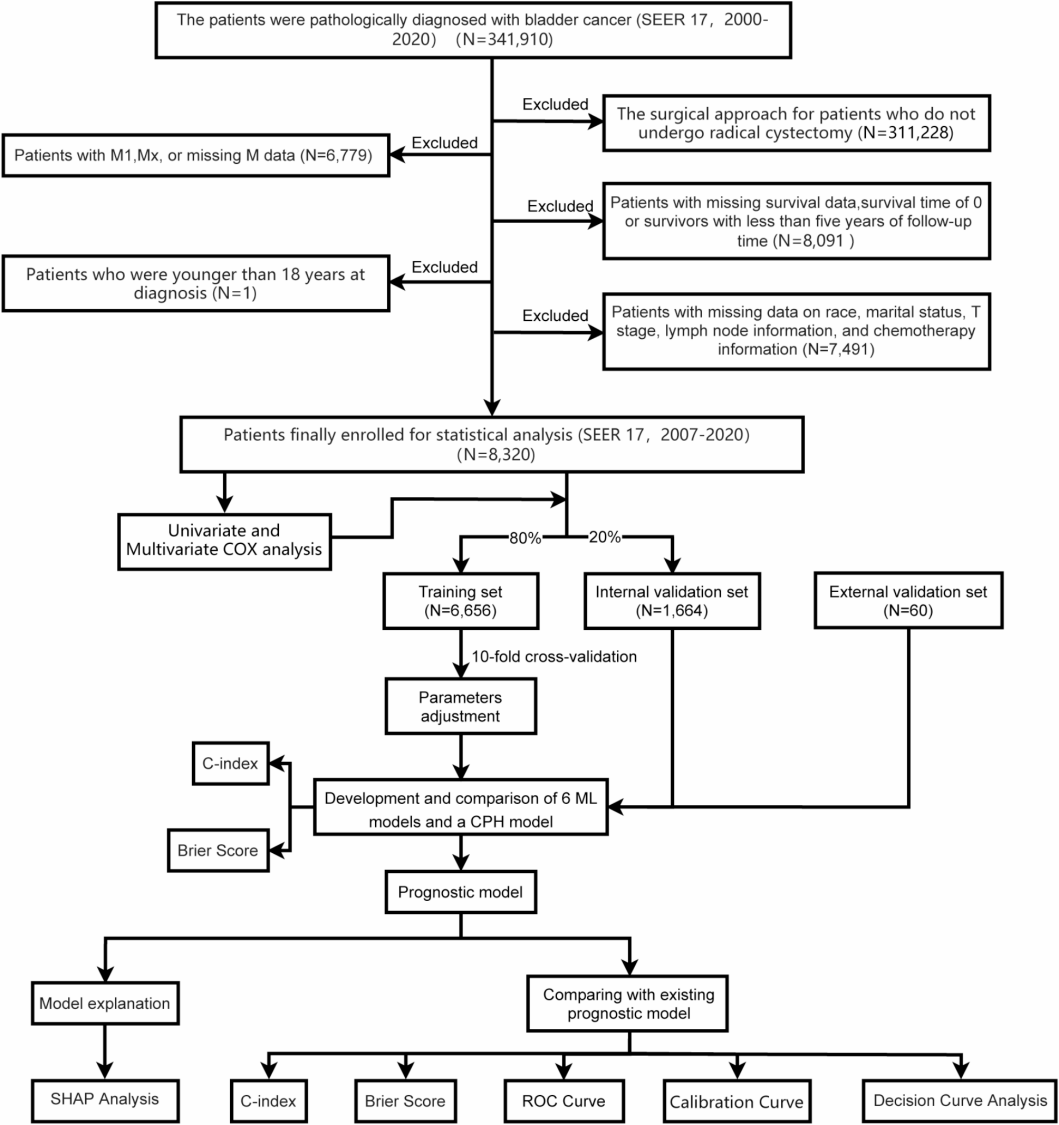
Flowchart of patient selection and study design. Legend: SEER: Surveillance, Epidemiology, and End Results; M: metastasis; T: tumor; C-index: concordance index; ML: machine learning; CPH: Cox proportional hazard; SHAP: Shapley additive explanation; ROC: receiver operating characteristic

Figure 1 shows details of the study design. A total of 8,320 M0 patients who underwent radical cystectomy and LN dissection were identified and screened for additional analysis based on the inclusion criteria. These patients were randomly divided into training (6,656) and internal validation (1,664) sets in an 8:2 ratio. Additionally, we collected 60 samples from the First Affiliated Hospital of Sun Yat-sen University as an external validation set. Table 1 presents the baseline characteristics of the study cohort, including age (< 65, ≥ 65), sex (female, male), race (black, white, other), marital status (married, never married, SDW: separated, divorced or widowed), pathology (urothelial carcinoma, non-urothelial carcinoma), tumor size (< 30 mm, ≥ 30 mm), grade (I-II, III–IV), T stage (T1/Ta/Tis, T2, T3, T4), LN density (0, 0-0.33, 0.33–0.5, 0.5-1), chemotherapy (no, NAC: neoadjuvant therapy, CT: adjuvant therapy). In the training, internal validation, and external validation sets, the 5-year CSM rates of patients were 55.11%, 48.68%, and 48.33%, respectively. The median follow-up times for these sets were 58, 62, and 60 months, respectively.

**Table 1:** Baseline demographical and clinicopathological characteristics of patients

Characteristics	Training set	Internal validation set	External validation set
	(*N* = 6,656)	(*N* = 1,664)	(*N* = 60)
Age			
< 65	2,365 (35.53%)	584 (35.10%)	27 (45.00%)
≥ 65	4,291 (64.47%)	1,080 (64.90%)	33 (55.00%)
Sex			
Female	1,662 (24.97%)	396 (23.80%)	8 (13.33%)
Male	4,994 (75.03%)	1,268 (76.20%)	52 (86.67%)
Race			
Black	387 (5.81%)	107 (6.43%)	-
White	5,884 (88.40%)	1,461 (87.80%)	-
Other	385 (5.78%)	96 (5.77%)	60 (100.00%)
Marital status			
Married	4,346 (65.29%)	1,090 (65.50%)	60 (100.00%)
Never married	860 (12.92%)	203 (12.20%)	-
SDW	1,450 (21.78%)	371 (22.30%)	-
Pathology			
Urothelial carcinoma	5,995 (90.07%)	1,496 (89.90%)	58 (96.67%)
Non-urothelial carcinoma	661 (9.93%)	168 (10.10%)	2 (3.33%)
Tumor size			
< 30 mm	1,415 (21.26%)	353 (21.21%)	11 (18.33%)
≥ 30 mm	5,241 (78.74%)	1,311 (78.79%)	49 (81.67%)
Grade			
I-II	363 (5.45%)	115 (6.91%)	13 (21.67%)
III-IV	6,293 (94.55%)	1,549 (93.09%)	47 (78.33%)
T stage			
Ta/Tis/T1	828 (12.44%)	219 (13.16%)	12 (20.00%)
T2	2,299 (34.54%)	591 (35.52%)	21 (35.00%)
T3	2,333 (35.05%)	572 (34.38%)	14 (23.33%)
T4	1,196 (17.97%)	282 (16.95%)	13 (21.67%)
Lymph node density^1^			
0	4,838 (72.69%)	1,193 (71.69%)	43 (71.67%)
> 0-0.33	1,248 (18.75%)	334 (20.07%)	13 (21.67%)
≥ 0.33–0.5	194 (2.91%)	57 (3.43%)	-
≥ 0.5-1	376 (5.65%)	80 (4.81%)	4 (6.67%)
Chemotherapy			
No	4,180 (62.80%)	1,054 (63.34%)	52 (86.67%)
NAC	891 (13.39%)	211 (12.68%)	1 (1.67%)
CT	1,585 (23.81%)	399 (23.98%)	7 (11.67%)
Cancer-specific Mortality			
5years	3,335 (50.11%)	810 (48.68%)	29 (48.33%)
3years	2,935 (44.10%)	698 (41.95%)	21 (35.00%)
1year	1,378 (20.70%)	338 (20.31%)	10 (16.67%)
Median follow up months (25th–75th percentile)	58 (14–100)	62 (14–106)	60 (19–65)

SDW: separated, divorced or widowed; T: tumor; NAC: neoadjuvant therapy; CT: adjuvant therapy

^1^ Lymph node density is calculated as the total number of positive lymph nodes/total number of lymph nodes removed

### Identification of prognostic factors

We first conducted univariate Cox regression analysis on the SEER database to identify variables significantly affecting patients’ cancer-specific survival (CSS), including age, sex, race, marital status, pathology, tumor size, T stage, LN density, and chemotherapy. Subsequently, multivariate Cox regression analysis was performed to control for confounding factors and to identify independent predictor variables influencing CSS (Table 2). Ultimately, eight independent prognostic factors were identified. The results revealed that age ≥ 65 years, black race, non-urothelial carcinoma, tumor size ≥ 30 mm, higher T stage, and higher LN density were associated with worse CSS. Conversely, being married and undergoing chemotherapy were associated with better CSS.

**Table 2:** Univariate and multivariate Cox regression analysis of selected variables for cancer-specific survival in the SEER database

Characteristics	Univariate analysis		Multivariate analysis	
	HR (95% CI)	*p* value	HR (95% CI)	*p* value
Age				
< 65	Reference		Reference	
≥ 65	1.30 (1.30–1.40)	< 0.001*	1.31 (1.23–1.40)	< 0.001*
Sex				
Female	Reference		Reference	
Male	0.88 (0.82–0.94)	< 0.001*	1.05 (0.98–1.12)	0.176
Race				
Black	Reference		Reference	
White	0.67 (0.60–0.75)	< 0.001*	0.79 (0.70–0.89)	< 0.001*
Other	0.59 (0.50–0.70)	< 0.001*	0.68 (0.58–0.81)	< 0.001*
Marital status				
Married	Reference		Reference	
Never married	1.19 (1.09–1.30)	< 0.001*	1.19 (1.09–1.30)	< 0.001*
SDW	1.32 (1.23–1.41)	< 0.001*	1.14 (1.06–1.23)	< 0.001*
Pathology				
Urothelial carcinoma	Reference		Reference	
Non-urothelial carcinoma	1.40 (1.30–1.50)	< 0.001*	1.24 (1.12–1.37)	< 0.001*
Tumor size				
< 30 mm	Reference		Reference	
≥ 30 mm	1.40 (1.30–1.50)	< 0.001*	1.21 (1.12–1.31)	< 0.001*
Grade				
I-II	Reference		Reference	
III-IV	0.96 (0.85–1.10)	0.56	1.02 (0.90–1.17)	0.714
T stage				
Ta/Tis/T1	Reference		Reference	
T2	1.35 (1.19–1.53)	< 0.001*	1.31 (1.15–1.49)	< 0.001*
T3	3.56 (3.15–4.02)	< 0.001*	2.88 (2.54–3.26)	< 0.001*
T4	5.40 (4.76–6.14)	< 0.001*	3.92 (3.44–4.48)	< 0.001*
Lymph node density^1^				
0	Reference		Reference	
> 0-0.33	2.50 (2.34–2.68)	< 0.001*	2.05 (1.90–2.20)	< 0.001*
≥ 0.33–0.5	3.63 (3.16–4.16)	< 0.001*	2.70 (2.34–3.11)	< 0.001*
≥ 0.5-1	4.53 (4.08–5.03)	< 0.001*	3.21 (2.88–3.59)	< 0.001*
Chemotherapy				
No	Reference		Reference	
NAC	0.83 (0.75–0.91)	< 0.001*	0.80 (0.73–0.88)	< 0.001*
CT	1.13 (1.06–1.21	< 0.001*	0.69 (0.64–0.74)	< 0.001*

SDW: separated, divorced or widowed; T: tumor; NAC: neoadjuvant therapy; CT: adjuvant therapy

^1^ Lymph node density is calculated as the total number of positive lymph nodes/total number of lymph nodes removed

*p < 0.05, indicating statistical significance

### Model development and selection

Six ML models and a CPH model were constructed using the training dataset. The performance of these seven models was compared on internal and external validation sets using C-index and Brier score. Table 3 indicates that, whether in the internal validation set or the external validation set, the LightGBM model consistently demonstrated better discrimination and calibration ability (internal validation set: C-index = 0.723, Brier score = 0.191; external validation set: C-index = 0.791, Brier score = 0.13). Then, the accuracy and precision of this model were assessed using a confusion matrix. In the internal validation set, the accuracy was 0.718 and the precision was 0.705. In the external validation set, the accuracy was 0.867 and the precision was 0.920 (Supplementary Figure S1). Following this, based on the predictions of the LightGBM model, patients were stratified into predicted death and predicted survival groups for K-M analysis curve plotting. As depicted in the Supplementary Figure S2, there were significant differences in survival time between the two groups (*p* < 0.001), indicating that our model can effectively assess patients’ prognosis.

**Table 3:** Performance of prognostic models in the internal validation sets and external validation sets

Models	Internal validation set		External validation set	
	C-index	Brier score	C-index	Brier score
LightGBM	0.723	0.191	0.791	0.134
GBDT	0.723	0.191	0.790	0.134
XGBoost	0.721	0.192	0.787	0.134
DT	0.706	0.201	0.713	0.175
AdaBoost	0.720	0.247	0.786	0.245
KNN	0.712	0.196	0.777	0.151
CPH	0.719	0.195	0.783	0.149

C-index: concordance index; LightGBM: Light Gradient Boosting Machine; GBDT: Gradient Boosting Decision Tree; XGBoost: Extreme Gradient Boosting; DT: Decision Tree; AdaBoost: Adaptive Boosting; KNN: K-Nearest Neighbor; CPH: Cox Proportional Hazard

### Model explanation

**Fig. 2:**
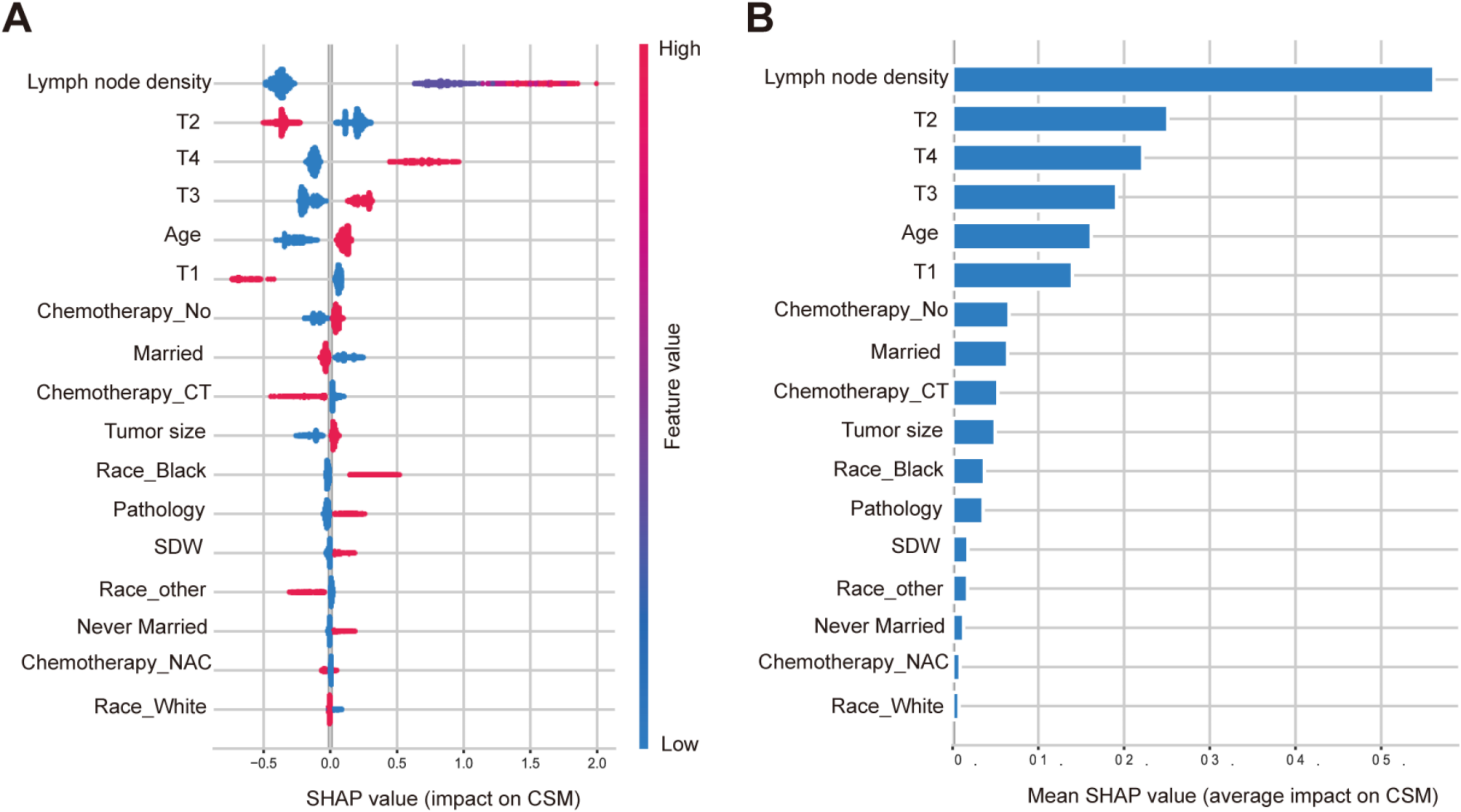
Global model explanation by the SHAP method. Legend: (**A**) SHAP summary dot plot. The probability of 5-year CSM increases with the SHAP value of a feature. Each patient in the model is represented by a dot on the line for each feature, with the color of the dots reflecting the actual feature values—red indicating higher values and blue indicating lower values. The dots are stacked vertically to show density. (**B**) SHAP summary bar plot. SHAP: Shapley additive explanation; CSM: cancer-specific mortality; SDW: separated, divorced or widowed; T: tumor; NAC: neoadjuvant therapy; CT: adjuvant therapy

**Fig. 3:**
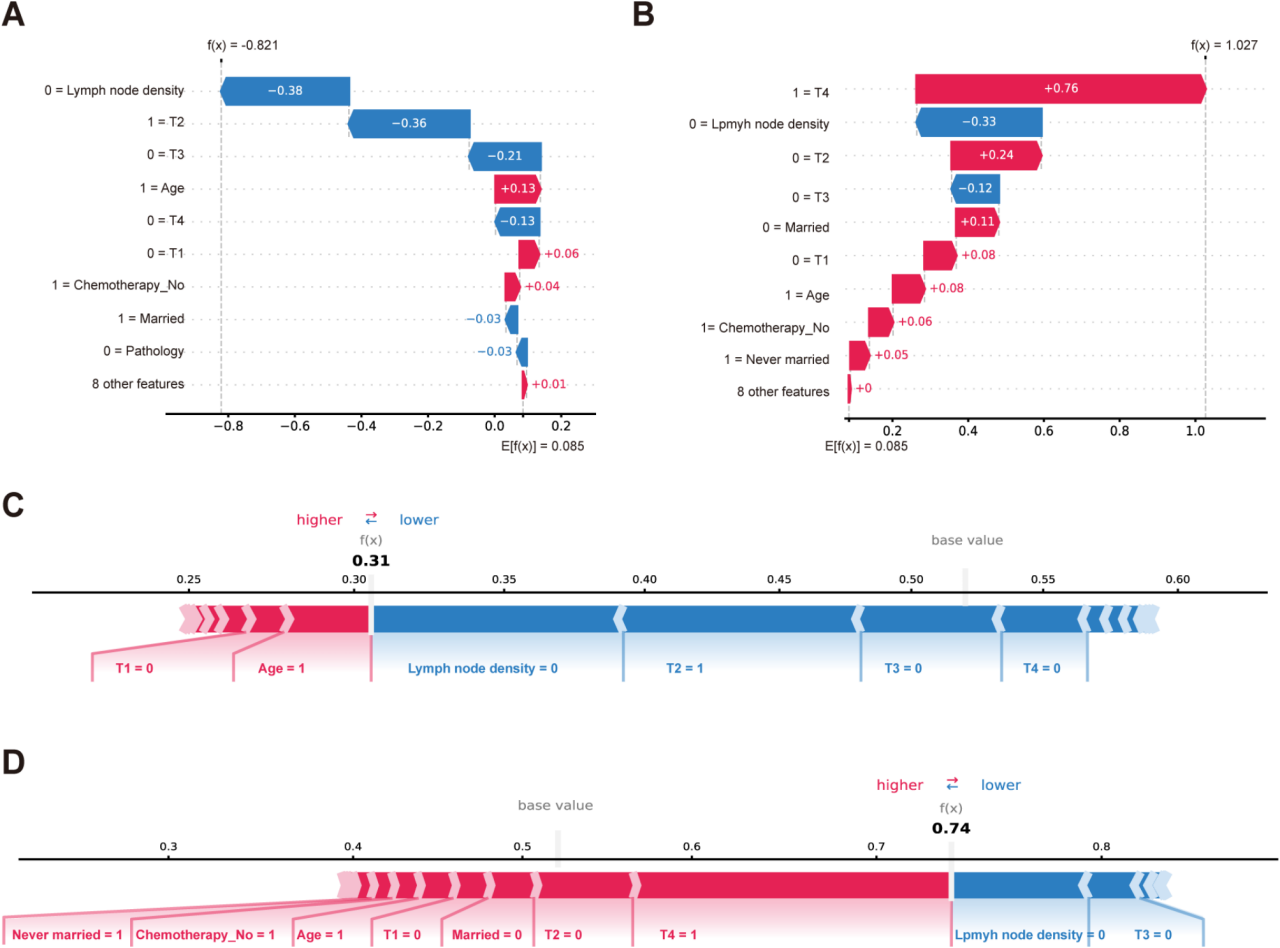
Local model explanation by the SHAP method. Legend: (**A**–**D**) are the waterfall and force plots for two individual patients. (**A**) and (**C**) represent individual patients classified under the “Survival” class, while (**B**) and (**D**) represent individual patients classified under the “Death” class. Blue features contribute to the prediction towards the “Survival” class, whereas red features contribute to the prediction towards the “Death” class. The waterfall plot outputs the SHAP values, and the force plot outputs the 5-year CSM. (**A**) and (**C**) output the patient’s SHAP value as -0.821, with a 5-year CSM of 31%; (**B**) and (**D**) output the patient’s SHAP value as 1.027, with a 5-year CSM of 74%. SHAP: Shapley additive explanations; CSM: cancer-specific mortality; T: tumor

Machine learning models are often perceived as black box models, making it challenging to understand their internal mechanisms. To enhance the model’s interpretability, we conducted SHAP analysis, providing two types of explanations: global explanations at the feature level and local explanations at the individual level. As shown in the SHAP summary plot (Fig. 2A-B), features were evaluated for their contributions to the model using average SHAP values, displayed in descending order. Additionally, local interpretation analyzes how personalized input data combine to make specific predictions for individuals. Figure 3 demonstrates the model’s personalized predictions for two specific patients. The waterfall plot (Fig. 3A-B) illustrates the contribution of each feature to the outcome, where blue indicates features that push the decision towards the ‘survival’ class, and red indicates features that push the decision towards the ‘death’ class, along with the corresponding SHAP values for each patient. In Fig. 3A, the SHAP value for the first patient is -0.821, while in Fig. 3B, the SHAP value for another patient is 1.027. Similarly, in the force plot (Fig. 3C-D), the blue features on the right side of the plot predict ‘survival’, while the red features on the left side predict ‘death’, resulting in predicted mortality rates of 0.31 for the first patient and 0.74 for the second patient.

### Model comparison

**Fig. 4:**
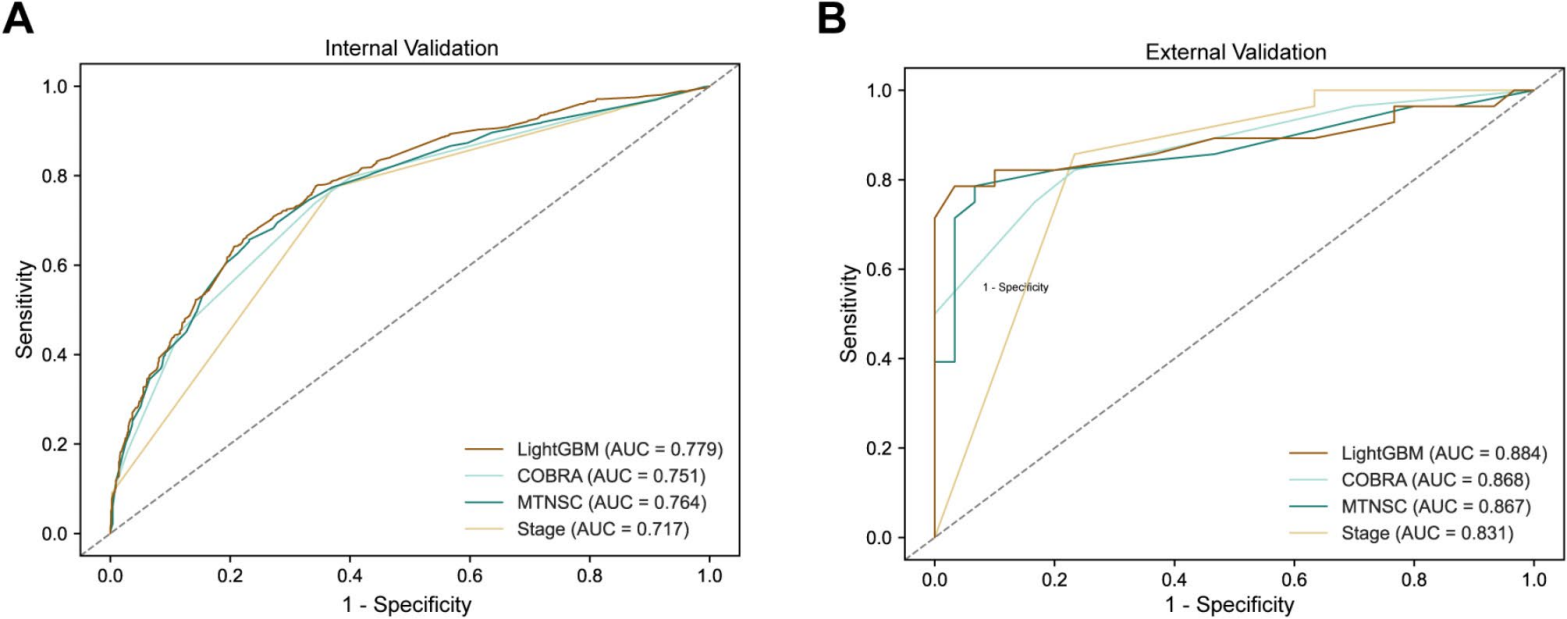
Comparison of ROC curves for multiple models. Legend: ROC curves for predicting 5-year CSM in urothelial carcinoma patients after radical cystectomy. (**A**) ROC curve for the internal validation set. (**B**) ROC curve for the external validation set. ROC: receiver operating characteristic; AUC: area under the curve; CSM: cancer-specific mortality; LightGBM: Light Gradient Boosting Machine; Stage: American Joint Committee on Cancer 8th edition stage; COBRA: Cancer of the Bladder Risk Assessment; MTNSC: Marital status, Tumor, Nodes, Size, Chemotherapy

**Fig. 5:**
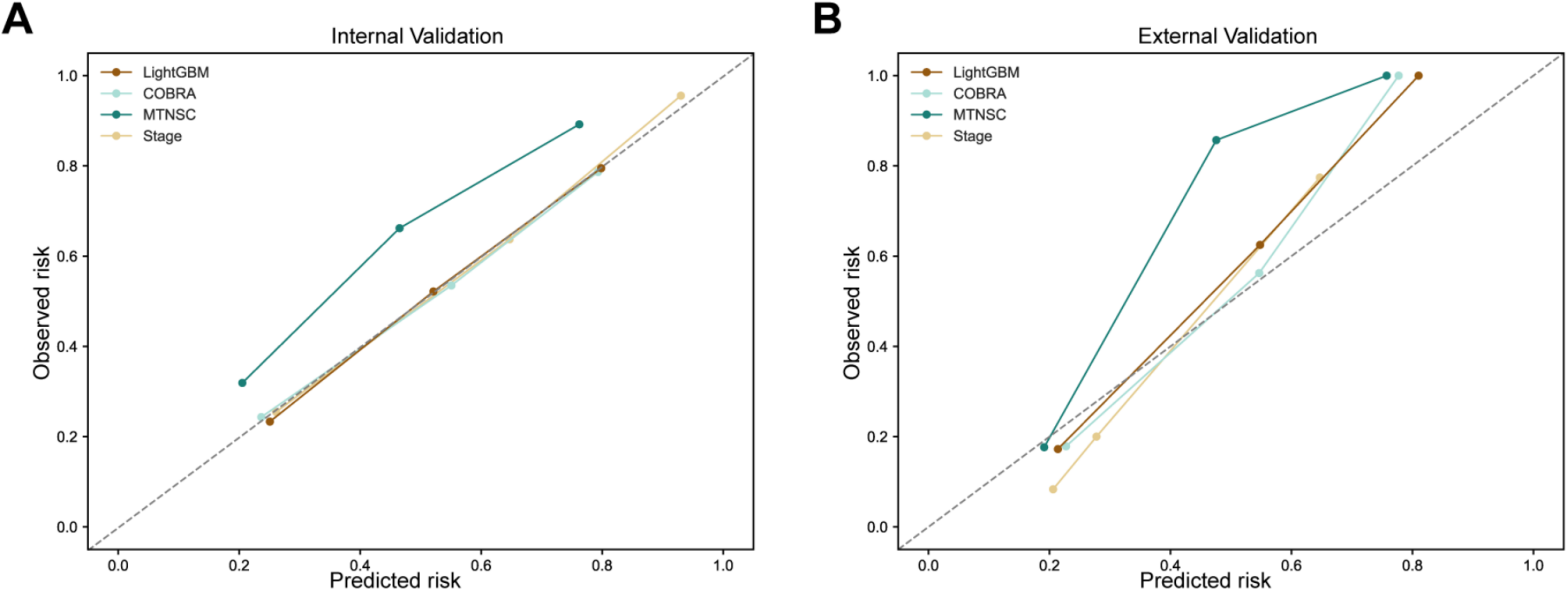
Comparison of calibration curves for multiple models. Legend: Calibration curves for predicting 5-year CSM in urothelial carcinoma patients after radical cystectomy. (**A**) Calibration curve for the internal validation set. (**B**) Calibration curve for the external validation set. CSM: cancer-specific mortality; LightGBM: Light Gradient Boosting Machine; Stage: American Joint Committee on Cancer 8th edition stage; COBRA: Cancer of the Bladder Risk Assessment; MTNSC: Marital status, Tumor, Nodes, Size, Chemotherapy

**Fig. 6:**
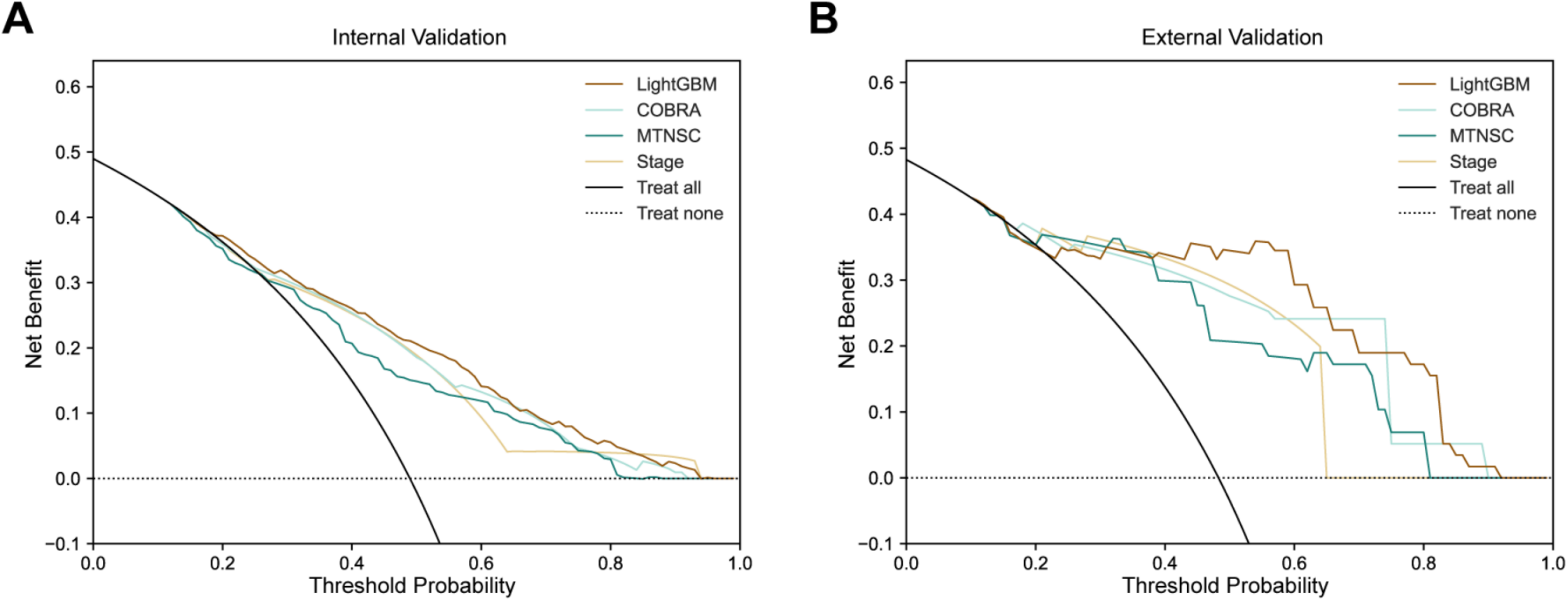
Decision curve analysis for multiple models. Legend: Decision curve analysis for predicting 5-year CSM in urothelial carcinoma patients after radical cystectomy. (**A**) Decision curve analysis for the internal validation set. (**B**) Decision curve analysis for the external validation set. CSM: cancer-specific mortality; LightGBM: Light Gradient Boosting Machine; Stage: American Joint Committee on Cancer 8th edition stage; COBRA: Cancer of the Bladder Risk Assessment; MTNSC: Marital status, Tumor, Nodes, Size, Chemotherapy

Compared to the three existing prognostic models in urothelial carcinoma patients, Fig. 4; Table 4 demonstrate that LightGBM model (internal validation set: C-index = 0.718, AUC = 0.779, Brier score = 0.191; external validation set C-index: 0.789, AUC = 0.884, Brier score = 0.137) outperformed the stage model (internal validation set: C-index = 0.665, AUC = 0.717, Brier score = 0.204; external validation set C-index: 0.741, AUC = 0.831, Brier score = 0.164), COBRA model (internal validation set: C-index = 0.697, AUC = 0.751, Brier score = 0.198; external validation set C-index: 0.776, AUC = 0.868, Brier score = 0.151), and MTNSC model (internal validation set: C-index = 0.706, AUC = 0.764, Brier score = 0.212; external validation set C-index: 0.780, AUC = 0.867, Brier score = 0.167). As shown in Fig. 5, the calibration curve also intuitively demonstrates the accuracy of each model. This indicates that our model has superior discrimination and calibration ability, as evidenced by its higher C-index and AUC values, and lower Brier score on both the internal and external validation sets. Subsequently, we evaluated the performance of the model by employing DCA, taking into account its impact on treatment decisions. Among the four models, the LightGBM model’s DCA curves showed larger net benefits across a wide range of threshold probabilities in both the internal and external validation sets, indicating that our model has superior clinical utility compared to the other three models (Fig. 6).

**Table 4:** Comparison of each model at predicting 5-year cancer-specific mortality of patients with urothelial carcinoma

Models	Internal validation set		External validation set	
	C-index	Brier score	C-index	Brier score
LightGBM	0.718	0.191	0.789	0.137
Stage	0.665	0.204	0.741	0.164
COBRA	0.697	0.198	0.776	0.151
MTNSC	0.706	0.212	0.780	0.167

C-index: concordance index; LightGBM: Light Gradient Boosting Machine; Stage: American Joint Committee on Cancer 8th edition stage; COBRA: Cancer of the Bladder Risk Assessment; MTNSC: Marital status, Tumor, Nodes, Size, Chemotherapy

## Discussion

In this study, we developed and validated an explainable prognostic model using ML techniques to predict the 5-year CSM rate after radical cystectomy in bladder cancer patients. Moreover, we compared the performance of our model against three existing prediction models. To our knowledge, our study is the first to apply ML methods to the SEER database for predicting CSM in patients undergoing radical cystectomy. We observed that our model based on clinicopathological variables performed better than the 8th edition AJCC staging model [17], the COBRA score mode [9], and the MTNSC model [11].

Our predictive model incorporates eight variables: age, race, marital status, pathology, tumor size, T stage, LN density, and chemotherapy. All these variables were selected as independent prognostic markers through both univariate and multivariate Cox regression analysis. As shown in Fig. 2 of the SHAP analysis, LN density and T stage have the most significant impact on prognosis in our model. Higher LN density and T stage often indicate poorer prognosis, consistent with previous research findings [18, 19]. Previous studies have also suggested that neoadjuvant chemotherapy, adjuvant chemotherapy, being married, and smaller tumor volume are favorable prognostic factors [20–24], our study results confirm these findings. Additionally, we observed that non-urothelial carcinoma and Black race are adverse prognostic factors, as depicted in Fig. 2; Table 2.

The AJCC staging system is one of the widely used tumor staging systems in clinical practice, which includes primary tumor (T) status, regional lymph node (N), and distant metastasis (M), providing accurate prognostic assessment for patients. The COBRA score was developed to predict CSM after surgery and was based on the SEER cancer database. The COBRA score, which incorporates age, tumor stage, and LN density, has demonstrated good performance in predicting CSM in bladder cancer patients and has been externally validated in multiple studies [25–27]. The MTNSC model, also based on the SEER database, includes variables such as marital status, T stage, N stage, primary tumor size, and chemotherapy. Unlike the previous two models, the MTNSC model is a multivariate model. There are also several pre-existing models that have been constructed and externally validated [7, 12, 28–31], but they were not compared with our model as they include variables such as lymphovascular invasion, which may not be routinely available from all pathology reports, limiting their usability.

From the comparison of the four models, our model performs the best in terms of discrimination and calibration ability, with the highest C-index, AUC, and the lowest Brier score. The Stage model shows the poorest performance, which is consistent with previous research findings [11, 27]. Compared to the COBRA model, the MTNSC model exhibits better discrimination ability but poorer calibration. As Fig. 6 depicted in the DCA curve, our model demonstrates greater net benefit across a wide range of threshold probabilities in both internal and external validation sets, indicating its superior clinical utility compared to the other three models. Additionally, our model is applicable to bladder cancer patients with non-urothelial carcinoma, whereas the COBRA score and MTNSC model are only applicable to patients with urothelial carcinoma.

With the abundance of electronic patient health data, ML offers a contemporary and precise method to effectively predict personalized survival outcomes, enabling improved decision-making capabilities [32]. It has been applied in various medical fields and has demonstrated impressive performance [14–16, 33]. However, there is a limited number of ML studies in bladder cancer that use clinicopathological data to predict the prognosis of bladder cancer patients. Bhambhvani et al. [34]used artificial neural networks to construct a 5-year prognosis model for bladder cancer using the SEER database; Eminaga et al. [35] also used machine learning methods to construct CSS prognosis models for urologic cancers. However, these studies suffer from the following limitations: (1) Lack of external validation. (2) All stages and surgical methods of patients were included without further stratification of bladder cancer patients, lacking specificity and having limited clinical significance.

Urothelial carcinoma is the most common type of bladder cancer, with subtypes such as micropapillary, plasmacytoid, small cell, and sarcomatoid variants, which differ in prognosis. Some subtypes, like the micropapillary variant, have poorer outcomes and higher recurrence rates after radical cystectomy [36]. For these special subtypes, trimodal therapy may serve as an alternative treatment option [37]. In our model, due to the limited number of samples for other subtypes, we combined non-urothelial carcinoma subtypes in the modeling process. By combining these subtypes, we can avoid overfitting or overlooking less frequent subtypes, making the model more balanced, improving predictive accuracy, and reducing the risk of overfitting. In addition, when constructing the model, we chose lymph node density instead of the number of positive LNs or traditional pN staging because LN density offers additional advantages. LN density not only considers the number of positive LNs but also accounts for the total number of LNs removed, providing a more comprehensive assessment of the patient’s lymph node status. Compared to relying solely on the number of positive LNs or pN staging, LN density offers a more precise measure, enabling finer risk stratification among patients and resulting in more accurate prognostic evaluation [38]. This approach effectively minimizes bias caused by differences in the extent of lymph node dissection, leading to more stable and reliable predictive outcomes.

Our ML model was trained using real-world data from a high-quality database that included a diverse and contemporary population with various ethnic backgrounds, enhancing the model’s applicability to a broad range of people. We employed a comprehensive methodology that involved evaluating discrimination, calibration, and clinical effectiveness. However, this study has some important limitations. Firstly, our study is based on SEER data, which is susceptible to coding errors, incomplete data collection, and inaccuracies. Secondly, we excluded patients with missing information regarding race, marital status, T stage, total lymph node counts, positive lymph node counts, M stage, and chemotherapy status, and used multiple imputation methods to address missing data for tumor size and grade, which may introduce bias. Moreover, due to the limitations of the SEER database, we were unfortunately unable to include several factors that could potentially have a significant impact on prognosis, which in turn affected the comprehensiveness and accuracy of the model. One important factor that was not included is the surgical margin status and location. Research has shown that patients with postoperative positive surgical margins tend to have a poor prognosis, with approximately half of them experiencing recurrence or death within one year [39]. Moreover, the disease-specific mortality rate is higher in patients with positive surgical margins in the urethra and soft tissues compared to those with positive margins in the ureter [40]. Since the SEER database does not provide relevant data, we were unable to use this as an inclusion or exclusion criterion to filter patients or incorporate it as a prognostic factor in our model. In addition, factors such as preoperative laboratory test results, lymphovascular invasion, and comorbidities are also closely related to prognosis. Beyond the aforementioned clinical and pathological features, the genomic patterns of bladder cancer are also associated with different clinical outcomes. Russo et al. found that Y chromosome loss (LoY) may serve as a biomarker of genomic instability, potentially affecting prognosis [41]. If these variables can be incorporated in future research, they are expected to significantly improve the model’s performance, thereby providing more accurate guidance for the prognosis prediction of bladder cancer patients. Finally, due to the retrospective nature of our study, the results should be interpreted with caution. While we utilized an external validation cohort to validate the model, the sample size of our model remains relatively small, necessitating validation in larger external cohorts to ensure the reproducibility of its performance, as well as further prospective studies to validate our conclusions.

## Conclusions

This study used a large number of cases with demographic information, clinical-pathologic factors, and treatment features, successfully developing an explainable ML model to predict the 5-year CSM after radical cystectomy in bladder cancer patients. Furthermore, compared to existing models, our model demonstrated better discrimination and calibration ability. However, large prospective clinical studies are needed for external validation. Machine learning’s ability to handle vast amounts of data may provide numerous future advantages, including its potential to readily incorporate new data, self-train, and adapt to evolving input variables. Finally, its capacity to discern links between data could significantly improve our understanding of cancer evolution.

## Electronic supplementary material

Below is the link to the electronic supplementary material.

Supplementary Material 1: Additional file 1: Table S1. Comparing Variables Included in Prognostic Models. Table S2. Information on the methodology and statistical packages used in this work. Figure S1. Confusion matrix of the LightGBM model’s predicted results. Figure S2. Kaplan-Meier survival analysis. Figure S3. Performance of the LightGBM model.

## Data Availability

The data for model training and internal validation in the current study is openly available in the SEER database (https://seer.cancer.gov/). The external validation data for this study are available on request to the corresponding author.

## Abbreviations

LN: Lymph node
CSM: Cancer-specific mortality
ML: Machine learning
SEER: Surveillance, Epidemiology, and End Results
T: Tumor
M: Metastasis
LightGBM: Light Gradient Boosting Machine
GBDT: Gradient Boosting Decision Tree
XGBoost: Extreme Gradient Boosting
DT: Decision Tree
AdaBoost: Adaptive Boosting
KNN: K-Nearest Neighbor
CPH: Cox Proportional Hazards
C-index: Concordance index
K-M: Kaplan-Meier
SHAP: Shapley additive explanations
AJCC: American Joint Committee on Cance
COBRA: Cancer of the Bladder Risk Assessment
MTNSC: Marital status, Tumor, Nodes, Size, Chemotherapy
ROC: Receiver operating characteristic
AUC: Area under the curve
DCA: Decision curve analysis
SDW: Separated, divorced or widowed
NAC: Neoadjuvant therapy
CT: Adjuvant therapy
CSS: Cancer-specific survival
