# Supplementary material for: Using machine learning for predicting cancer-specific mortality in bladder cancer patients undergoing radical cystectomy: a SEER-based study
Source: BMC Cancer. 2025 Mar 21;25:523. doi: 10.1186/s12885-025-13942-2 (PMC11929216; doi:10.1186/s12885-025-13942-2)
Supplement: Supplementary file 1 — Supplementary Material 1: Additional file 1: Table S1. Comparing Variables Included in Prognostic Models. Table S2. Information on the methodology and statistical packages used in this work. Figure S1. Confusion matrix of the LightGBM model’s predicted results. Figure S2. Kaplan-Meier survival analysis. Figure S3. Performance of the LightGBM model. [file 12885_2025_13942_MOESM1_ESM.docx]

**Supplementary Table S1** Comparing Variables Included in Prognostic Models

|  | **Age** | **Race** | **Married** | **Pathology** | **Tumor** | **T** | **N stage or Lymph** | **M** | **Chemotherapy** |
| --- | --- | --- | --- | --- | --- | --- | --- | --- | --- |
|  |  |  |  |  | **Size** | **stage** | **node density** | **stage** |  |
| LightGBM | √ | √ | √ | √ | √ | √ | √ |  | √ |
| Stage |  |  |  |  |  | √ | √ | √ |  |
| COBRA | √ |  |  |  |  | √ | √ |  |  |
| MTNSC |  |  | √ |  | √ | √ | √ |  | √ |

T: tumor; N: nodes; M: metastasis; LightGBM: Light Gradient Boosting Machine; Stage: American Joint Committee on Cancer 8th edition stage; COBRA: Cancer of the Bladder Risk Assessment; MTNSC: Marital status, Tumor, Nodes, Size, Chemotherapy.

**Supplementary Table S2** Information on the methodology and statistical packages used in this work

|  | **Program** | **Package** |
| --- | --- | --- |
| Variable selection |  |  |
| Univariate Cox regression | R | survival |
| Multivariate Cox regression | R | survival |
| Model development |  |  |
| LightGBM | Python | Lightgbm |
| GBDT | Python | sklearn.ensemble  (GradientBoostingClassifier) |
| XGBoost | Python | Xgboost |
| DT | Python | sklearn.tree  (DecisionTreeClassifier) |
| AdaBoost | Python | sklearn.ensemble  (AdaBoostClassifier) |
| KNN | Python | sklearn.neighbors  (KNeighborsClassifier) |
| CPH | Python | Lifelines  (CoxPHFitter) |
| Model explanation |  |  |
| SHAP | Python | shap |
| Performance measure |  |  |
| C-index | Python | lifelines.utils  (concordance_index) |
| Brier score | Python | sklearn.metrics  (brier_score_loss) |
| ROC curve | Python | sklearn.metrics  (roc_curve, auc) |
| Calibration curve | Python | sklearn.calibration  (calibration_curve) |
| Confusion matrix | Python | sklearn.metrics  (confusion_matrix) |
| DCA | Python |  |
| K-M curve | Python | lifelines  （KaplanMeierFitter） |

LightGBM: Light Gradient Boosting Machine; GBDT: Gradient Boosting Decision Tree; XGBoost: Extreme Gradient Boosting; DT: Decision Tree; AdaBoost: Adaptive Boosting; KNN: K-Nearest Neighbor; CPH: Cox

Proportional Hazard; SHAP: Shapley additive explanations; C-index: Concordance index; ROC: receiver operating characteristic; DCA: decision curve analysis; K-M: Kaplan-Meier.


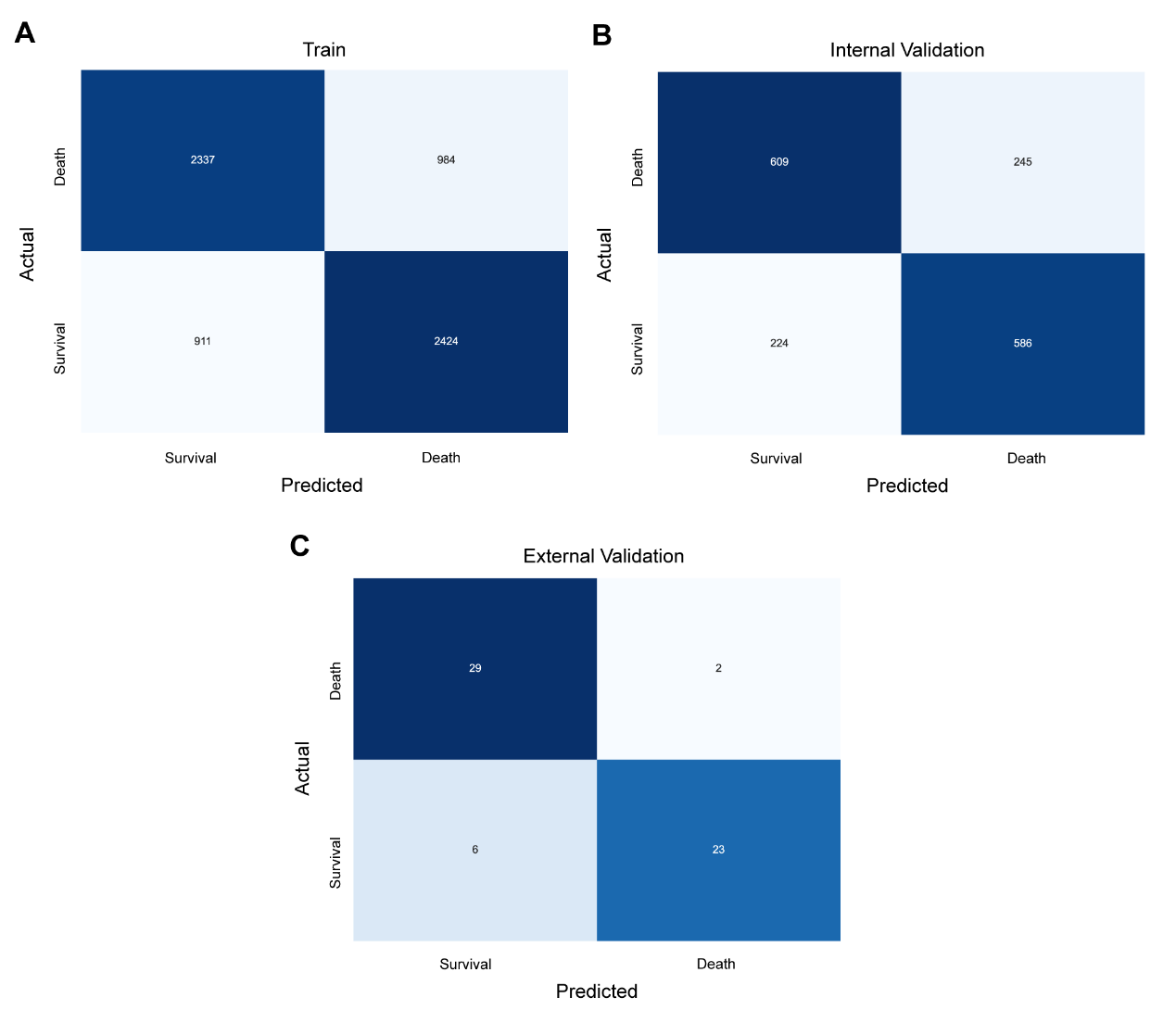


**Supplementary** **Figure S1:** **Title:** Confusion matrix of the LightGBM model’s predicted results. **Legend:** Confusion matrix of the LightGBM model to predict 5-year CSM of patients with bladder cancer after RC. (A) Training set. (B) Internal validation set. (C) External validation set. LightGBM: Light Gradient Boosting Machine; CSM: cancer-specific mortality; RC: radical cystectomy.


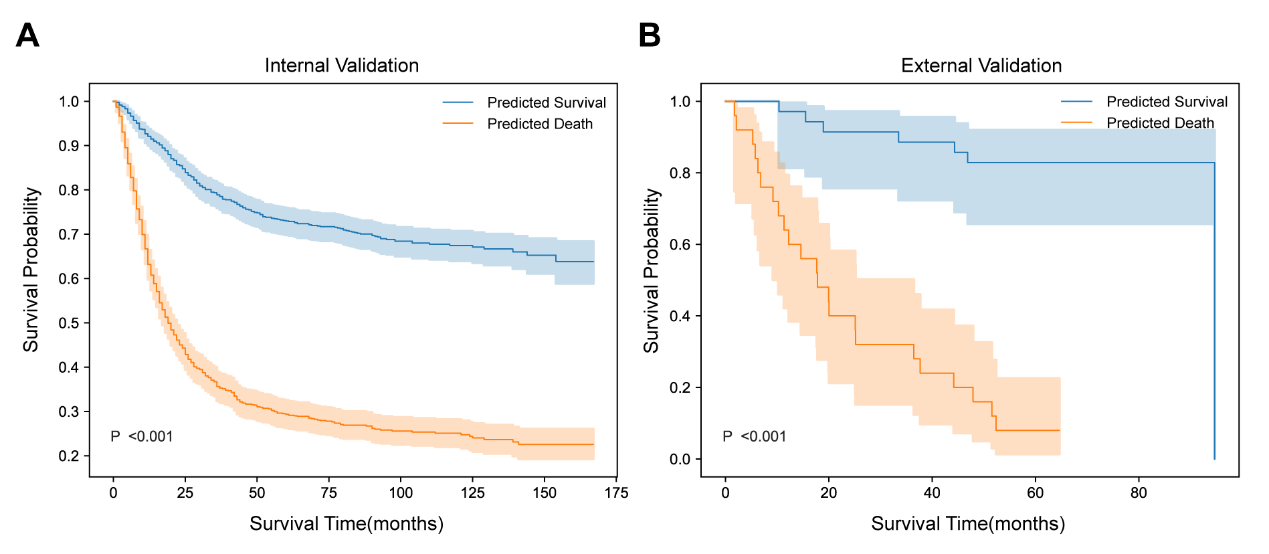


**Supplementary Figure S2:** **Title:** Kaplan-Meier survival analysis. **Legend:** Kaplan-Meier plots for describing cancer-specific survival in patients after RC stratified according to the LightGBM model’s predicted results. (A) Internal validation set. (B) External validation set. LightGBM: Light Gradient Boosting Machine; RC: radical cystectomy.


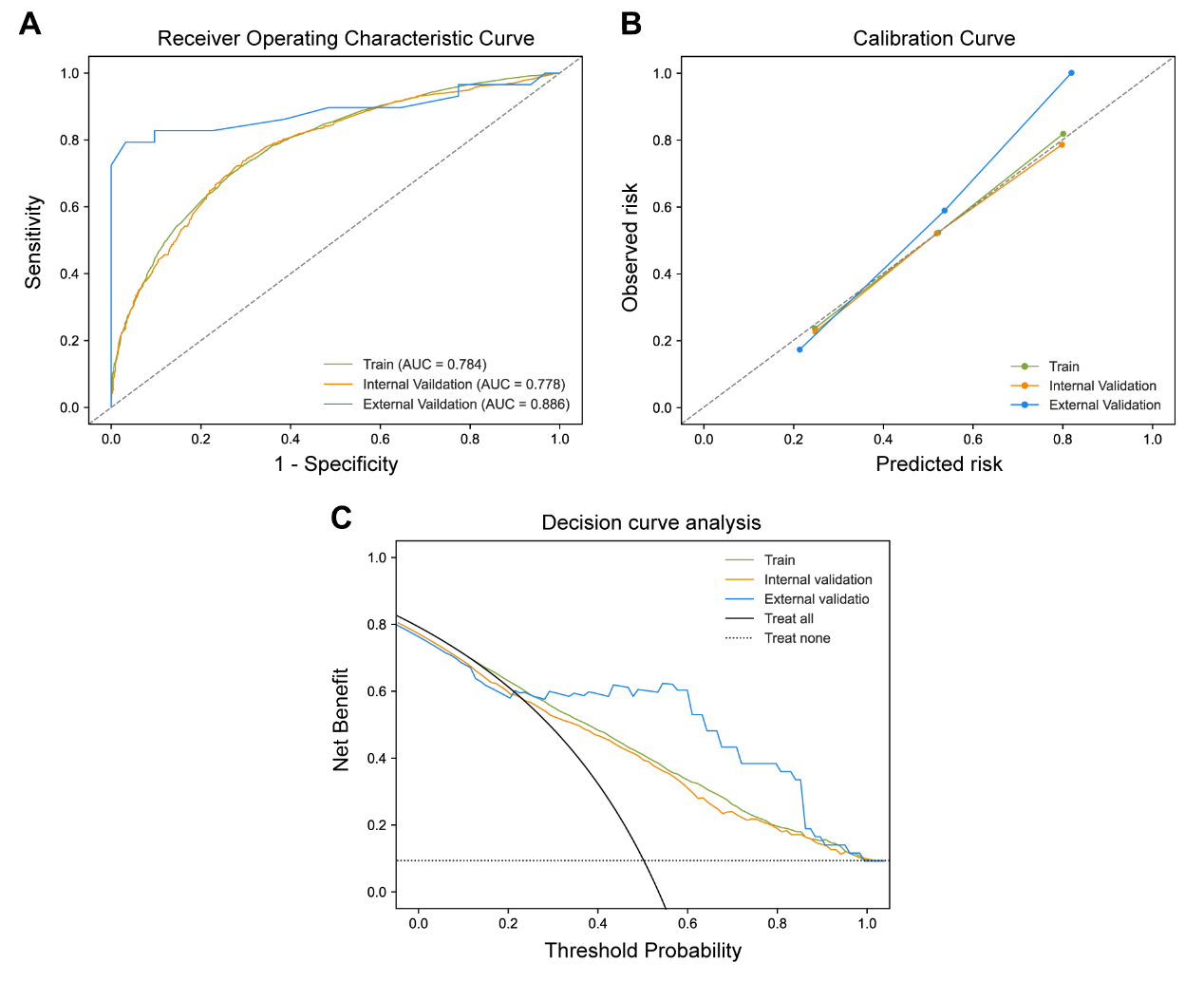


**Supplementary Figure S3: Title:** Performance of the LightGBM model. **Legend:** LightGBM model to predict 5-year CSM of patients with bladder cancer after RC. (A) ROC curve for the LightGBM model in the training, internal validation and external validation sets. (B) Calibration curve for the LightGBM model in the training, internal validation and external validation sets. (C) Decision curve analysis for the LightGBM model in the training, internal validation and external validation sets. AUC: area under the curve; LightGBM: light gradient boosting machine; CSM: cancer-specific mortality; RC: radical cystectomy.
